# Prognostic value of tumor measurement parameters and SCC-Ag changes in patients with locally-advanced cervical cancer

**DOI:** 10.1186/s13014-021-01978-0

**Published:** 2022-01-10

**Authors:** Wenjuan Chen, Siyi Xiu, Xingyun Xie, Huiming Guo, Yuanji Xu, Penggang Bai, Xiaoyi Xia

**Affiliations:** Department of Radiation Oncology, Fujian Medical University Cancer Hospital, Fujian Cancer Hospital, No. 420 Fuma Road, Fuzhou, 350000 Fujian China

**Keywords:** Cervical cancer, Tumor diameter, Tumor volume, Tumor volume reduction rate, Squamous cell carcinoma antigen (SCC-Ag)

## Abstract

**Objective:**

To investigate the prognostic relevance of specific measurement parameters such as tumor diameter, tumor volume, tumor volume reduction rate (TVRR), and changes in the squamous cell carcinoma antigen (SCC-Ag) level in patients with locally-advanced cervical cancer (LACC) undergoing concurrent radiotherapy and chemotherapy.

**Methods:**

This was a retrospective study of 203 patients with stage IIA–IVA cervical squamous cell carcinoma who were newly diagnosed at our hospital between January 2011 and March 2015. Clinical data and pre-and post-treatment imaging information were collected and each parameter was calculated using 3DSlicer software. The pre/post-treatment tumor diameter (TD_pre/post_), tumor volume (TV_pre/post_), SCC-Ag (SCC_pre/post_), and TVRR, SCC-Ag reduction rate (SCCRR) were analyzed and their prognostic relevance evaluated.

**Results:**

The median follow-up was 69 months. The 5-year overall survival (OS) and disease progression-free survival (PFS) rates were 69.5% and 64.5%, respectively. On univariate analysis, TD_pre/post_, TV_pre/post_, TVRR, SCC_pre/post_ and SCCRR showed significant association with OS and PFS (*P* < 0.05). On multivariate analysis, TD_pre_ [Hazard ratio (*HR)* = 0.373, *P* = 0.028], TD_post_ (*HR* = 0.376, *P* = 0.003) and SCC_post_ (*HR* = 0.374, *P* = 0.001) were independent predictors of OS. TVRR (*HR* = 2.998, *P* < 0.001), SCC_pre_ (*HR* = 0.563, *P* = 0.041), and SCC_post_ (*HR* = 0.253, *P* < 0.001) were independent predictors of PFS. Tumor measurement parameters showed a positive correlation with SCC-Ag (*P* < 0.05).

**Conclusion:**

TD_pre/post_, TV_pre/post_, TVRR, SCC_pre/post_, and SCCRR were prognostic factors in LACC. TD_pre/post_ and SCC_post_ showed the most significant prognostic value. TVRR and SCC_pre/post_ were closely related to disease progression. Further studies should investigate the correlation between measurement parameters of tumor and SCC-Ag.

## Background

Cervical cancer (CC) is the fourth most common malignant tumor in women. An estimated 530,000 new cases of CC and 270,000 deaths attributed to CC are reported each year across the world [1]. More than two-thirds of patients with CC have the locally-advanced disease at the time of diagnosis [2]. Concurrent chemoradiation is still the standard treatment for locally-advanced cervical cancer (LACC) [3]. The combination of external beam radiotherapy (EBRT) and brachytherapy (BRT) represents the mainstay in the primary treatment of patients with cervical cancer. While in elderly patients who refuse brachytherapy or are not amenable to brachytherapy, intensity modulated radiation therapy with simultaneous integrated boost (SIB) to macroscopic disease can be proposed, as an alternative to brachytherapy [4]. Studies have demonstrated the prognostic value of clinical stage, pathological type, lymph node metastasis, depth of tumor invasion, tumor size, and tumor differentiation in patients with CC [5–7]. Tumor volume has always been a key determinant of the prognosis of CC [8, 9]. Squamous cell carcinoma antigen (SCC-Ag) is a protein (molecular weight: 48000d) which is often increased in patients with cervical squamous cell carcinoma [10]. Studies have shown that the change in SCC-Ag level is not only related to the tumor size, but also one of the important diagnostic and prognostic markers of CC [11–13].

The reported 5-year overall survival (OS) rate of patients with the International Federation of Gynecology and Obstetrics (FIGO) stage II, stage III, and stage IV CC are 65–69%, 40–43%, and 15–20%, respectively [14]. In recent years, several studies have investigated the prognostic value of several factors (such as tumor size, volume, lymph node status) and changes in SCC-Ag in predicting the treatment outcomes of patients with CC. SCC-Ag was shown to be a marker for early diagnosis and post-treatment disease recurrence [15, 16].

Previous studies have investigated the value of tumor diameter, volume, and SCC-Ag in predicting the therapeutic response of CC during radiotherapy [17]. However, there is no clear consensus on the optimal cut-off value for parameters such as tumor diameter, volume, and tumor volume reduction rate (TVRR).

Moreover, most previous studies have not analyzed the relationships among the pre-treatment, post-treatment tumor diameter (TD_pre_, TD_post_), and pre-treatment tumor volume (TV_pre_). Furthermore, the prognostic relevance of post-treatment tumor volume (TV_post_) and TVRR is not well characterized in patients with CC. Few studies have addressed the prognostic relevance of SCC-Ag-related parameters such as pre-treatment SCC-Ag (SCC_pre_), post-treatment SCC-Ag (SCC_post_), and SCC-Ag reduction rate (SCCRR) during RT for CC. Further in-depth exploration of the prognostic value of tumor measurement parameters and SCC-Ag level in patients with CC is a key imperative.

## Materials and methods

### Study population

We retrospectively reviewed data pertaining to 203 patients with locally-advanced cervical squamous cell carcinoma who were newly diagnosed at our center between January 2011 and March 2015. Patients were staged using the 2009 version of FIGO staging system. All patients had complete medical history and MRI images, and were treated with concurrent chemoradiotherapy and individualized high-dose rate intracavitary brachytherapy.

### Acquisition of tumor measurement parameters

The 3D Slicer software [18] is a scalable medical image processing and visualization application platform. Pre-trearment MR means the Magnetic resonance imaging prior to chemotherapy and radiationtherapy. Post-treatment MR was underwent nearly the end of the EBRT. Pre- and post-treatment MR imaging data of 203 patients were imported into DICOM format and processed by the 3D Slicer software. Two radiologists delineated and outlined the primary tumor target area and residual tumor target area during radiotherapy. TD_pre_ and TD_post_ were measured by the related software modules, and then the TV_pre_, TV_post_, and TVRR were calculated by 3D Slicer.

TVRR = (TV_pre_-TV_post_)/TV_pre_ × 100% (the difference between TV_pre_ and TV_post_ divided by the percentage of TV_pre_).

### Treatment strategy

All patients received CCRT. Radiotherapy consisted of intensity-modulated radiotherapy (IMRT) or conventional 4-field box conformal radiotherapy technique (CRT). The external whole-pelvis irradiation was performed with a dose of 1.8–2.0 Gy per fraction 5 times per week up to a total external dose of 45.0–50.0 Gy. For positive pelvic lymph nodes, the radiotherapy dose was boosted to 10–16 Gy. This was followed by a high-dose rate intracavitary radiation with a fractional dose of 7.0 Gy (weekly) to a total dose of 28.0 Gy in four weeks. The preferred regimen in the guideline of National Comprehensive Cancer Network is cisplatin [3]. While many patients can not tolerant cisplatin because it is highly emetic and nephrotoxic. So chemotherapy was applied during radiotherapy, using nedaplatin monotherapy every three weeks at a dose of 80 mg/m^2^ or nedaplatin in combination with paclitaxel 135 mg/m^2^.

### Statistical analysis

The changes in each parameter (independent and dependent groups) were compared using *t* test. Kaplan–Meier method was used for survival analysis. Log-rank test and Cox proportional hazard regression model were applied to analyze the prognostic factors among parameters related to TD, TV, and SCC-Ag level. Receiver operating characteristic (ROC) curve analysis was performed to determine the optimal cut-off values using the Youden index. *P* values < 0.05 were considered indicative of statistical significance. All statistical analyses were performed using SPSS 26.0 (SPSS Inc., Chicago, Illinois).

## Results

### Characteristics of the study population

**Fig. 1:**
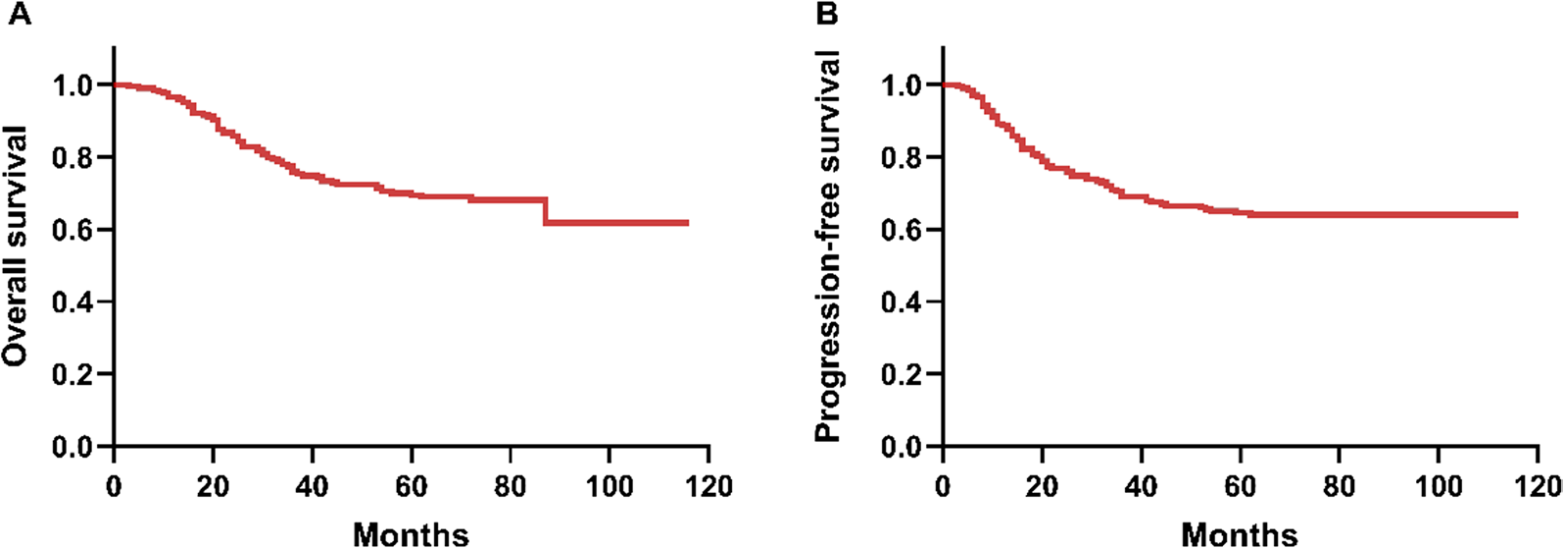
Survival analysis. **a** Overall survival (OS) and **b** progression-free survival (PFS)

The median age of patients in our cohort (n = 203) was 52 years (range, 32–76). The median interval between the pre-treatment and post-treatment MR was 45 days (range 35–71).The basic information and clinical characteristics of the study population are summarized in Table 1. The dicotimization value of age, TD_pre_, TV_pre_, TD_post_, TV_post_, TVRR, SCC_pre_ and SCC_post_ are based on the analysis of the ROC curves.It should be noticed that 80 patients (39.4%) underwent conventional radiation therapy due to poor economic conditions. The median duration of follow-up was 69 months (range 3–116). Among the 203 patients, 11 patients had local or regional recurrence; 28 patients had distant metastasis; and 3 patients had local/regional recurrence and distant metastasis at the same time. Among the 65 patients who died, 27 died of local regional recurrence or distant metastasis; 24 patients died of complications; and 14 patients died of unknown causes. The 5-year OS and PFS in our cohort were 69.5% and 64.5%, respectively (Fig. 1).

**Table 1:** Patient and tumor characteristics

Characteristics	n	%
Age (years)		
< 54	108	53.2
≥ 54	95	46.8
FIGO stage		
IIa	14	6.9
IIb	105	51.7
IIIa	8	3.9
IIIb	75	36.9
IVa	1	0.5
Infection		
Yes	15	7.4
No	188	92.6
Anemia		
Yes	80	39.4
No	123	60.6
Lymph node metastasis		
Yes	57	28.1
No	146	71.9
TD_pre_* (cm)		
≤ 4.4	88	43.3
> 4.4	115	56.7
TV_pre_* (cm^3^)		
≤ 45.71	105	51.7
> 45.71	98	48.3
TD_post_* (cm)		
≤ 2.4	127	62.6
> 2.4	76	37.4
TV_post_* (cm^3^)		
≤ 10.45	137	67.5
> 10.45	66	32.5
TVRR* (%)		
< 80.1	78	38.4
≥ 80.1	125	61.6
Radiotherapy		
IMRT	123	60.6
CRT	80	39.4
Total dose of radiotherapy (Gy)		
≤ 84	107	52.7
> 84	96	47.3
Number of chemotherapy cycles		
< 4	65	32.0
≥ 4	138	68.0
SCC_pre_ (µg/L)		
≤ 11.4	145	71.4
> 11.4	58	28.6
SCC_post_ (µg/L)		
≤ 1.9	180	88.7
> 1.9	23	11.3
SCCRR (%)		
= 100	122	60.1
< 100	24	11.8

*Radiological characteristics

### Analysis of tumor measurement parameters and SCC-Ag value

The median TD_pre_ and TD_post_ in our cohort were 4.5 cm (range, 1.7–9.7) and 2.1 cm (0.7–7.7), respectively; the median TV_pre_ and TV_post_ were 45.08 cm^3^ (range, 4.80–328.71) and 6.52 cm^3^ (0.41–140.45); the median TVRR was 0.84% (range, 0–0.98); the median SCC_pre_ and SCC_post_ were 4.7 µg/L (range, 0.5–70.0) and 0.9 µg/L (range, 0.2–47.8), respectively; and the median SCCRR was 1.0 (0–1.0) × 100%. Among the 203 patients included, pre-treatment SCC values of 57 patients were within the normal range (normal reference range: < 2 µg/L). In order to reduce statistical errors, the SCC values of these 57 patients were processed as missing values.

### ROC curve analysis

**Fig. 2:**
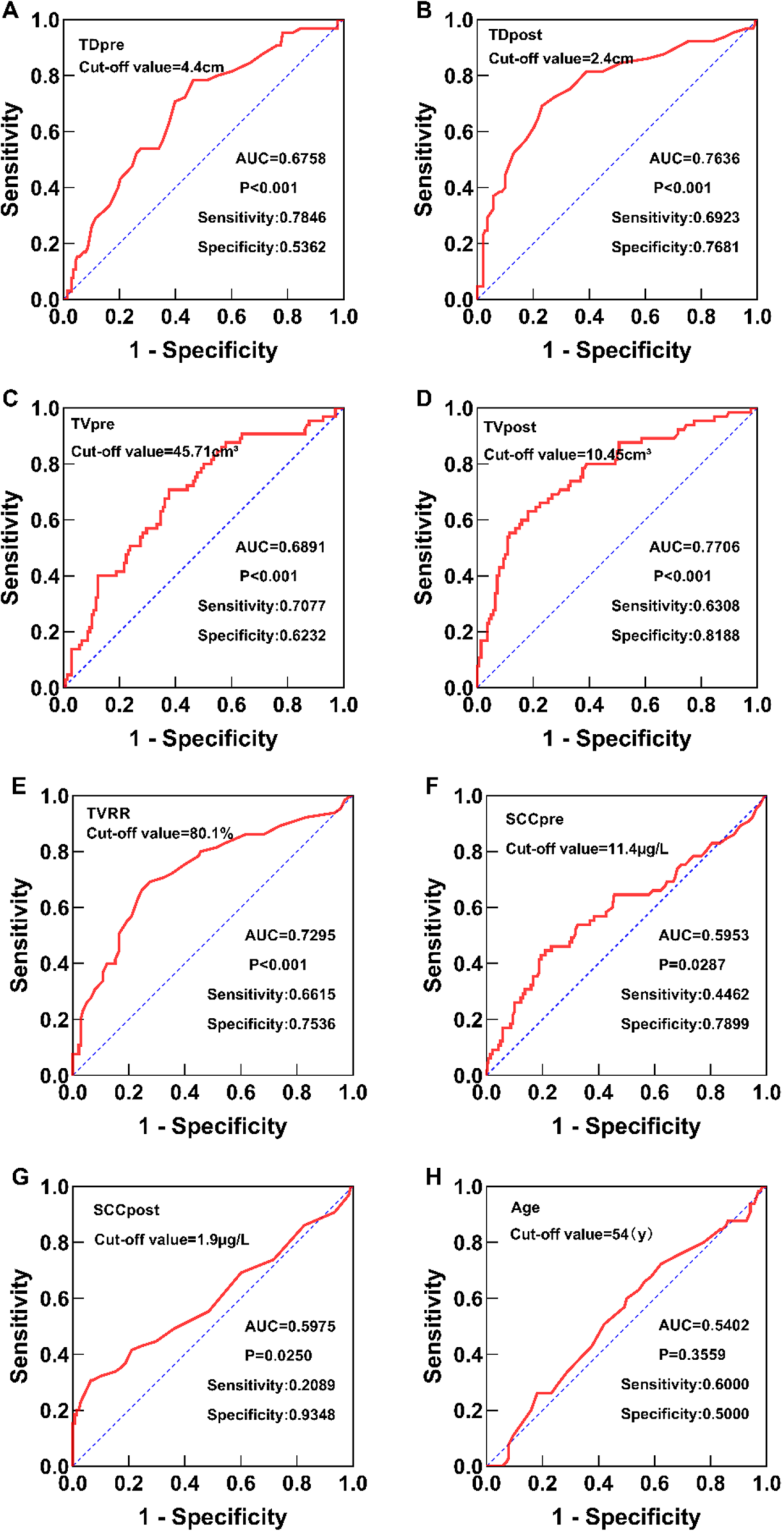
Results of receiver operating characteristic (ROC) curve analysis: **a** TD_pre_; **b** TD_post_; **c** TV_pre_; **d** TV_post_; **e** TVRR; **f** SCC_pre_; **g** SCC_post_; **h** age

On ROC curve analysis, the optimal cut-off value of TD_pre_ and TD_post_ (based on the Youden index) was 4.4 cm and 2.4 cm, respectively. The optimal cut-off value of TV_pre_ and TV_post_ was 45.71 cm^3^ and 10.45 cm^3^, respectively. The optimal cut-off value of TVRR was 80.1%. The optimal cut-off value for SCC_pre_ and SCC_post_ was 11.4 µg/L and 1.9 µg/L, respectively. The optimal cut-off value for age was 54 years (Fig. 2).

### Survival analysis

#### Analysis of OS

TDpre, TDpost, TVpre, TVpost, TVRR, SCCpre, SCCpost, SCCRR, FIGO staging, lymph node metastasis, and chemotherapy cycles all showed a significant association with OS (*P* < 0.05). The 5-year OS rate in the TD_pre_ ≤ 4.4 cm group was significantly greater than that in the TD_pre_ > 4.4 cm group (84.1% vs 58.3%, *P* < 0.001). The 5-year OS rate in the TV_pre_ ≤ 45.71 cm^3^ group was significantly greater than that in the TV_pre_ > 45.71 cm^3^ group (81.9% vs 56.1%, *P* < 0.001). The 5-year OS rate in the TD_post_ ≤ 2.4 cm group and TD_post_ > 2.4 cm group was 82.5% and 44.7%, respectively (*P* < 0.001). The 5-year OS rate in the TV_post_ ≤ 10.45 cm^3^ group and TV_post_ > 10.45 cm^3^ group was 82.5% and 42.4%, respectively (*P* < 0.001). The 5-year OS in the TVRR ≥ 80.1% group was also significantly greater than that in the TVRR < 80.1% group (84.0% vs 46.2%, *P* < 0.001). The 5-year OS rate in the SCC_pre_ ≤ 11.4 μg/L group and SCC_pre_ > 11.4 μg/L group was 75.2% and 55.2%, respectively (*P* = 0.001). The 5-year OS rate in the SCC_post_ ≤ 1.9 μg/L group and SCCpost > 1.9 µg/L was 75.3% and 34.5%, respectively (*P* < 0.001). The 5-year OS rates in the group with SCCRR of 100% and SCCRR < 100% were 75.4% and 33.3%, respectively (*P* < 0.001) (Table 2).

**Table 2:** Univariate analysis of OS and PFS

Variable	Univariate analysis			
	5-y OS (%)	*P*	5-y PFS (%)	*P*
Age (years)				
< 54	65.7	0.201	59.3	0.118
≥ 54	73.7		70.5	
FIGO stage				
≤ IIb	78.2	0.001	73.1	0.002
> IIb	57.1		52.4	
Infection				
Yes	33.3	< 0.001	33.3	0.001
No	72.3		67.0	
Anemia				
Yes	56.2	< 0.001	50.0	< 0.001
No	78.0		74.0	
Lymph node metastasis				
Yes	56.1	0.012	47.4	< 0.001
No	74.7		71.2	
TD_pre_* (cm)				
≤ 4.4	84.1	< 0.001	80.7	< 0.001
> 4.4	58.3		52.2	
TV_pre_* (cm^3^)				
≤ 45.71	81.9	< 0.001	78.1	< 0.001
> 45.71	56.1		50.0	
TD_post_* (cm)				
≤ 2.4	84.3	< 0.001	79.5	< 0.001
> 2.4	44.7		39.5	
TV_post_* (cm^3^)				
≤ 10.45	82.5	< 0.001	78.1	< 0.001
> 10.45	42.4		36.4	
TVRR* (%)				
< 80.1	46.2	< 0.001	41.0	< 0.001
≥ 80.1	84.0		79.2	
Radiotherapy				
IMRT	72.4	0.371	64.2	0.928
CRT	65.0		65.0	
Total dose of radiotherapy (Gy)				
≤ 84	71.0	0.783	62.6	0.497
> 84	67.7		66.7	
Number of chemotherapy cycles				
< 4	55.4	0.004	52.3	0.026
≥ 4	76.1		70.3	
SCC_pre_ (µg/L)				
≤ 11.4	75.2	0.001	73.1	< 0.001
> 11.4	55.2		43.1	
SCC_post_ (µg/L)				
≤ 1.9	75.3	< 0.001	70.6	< 0.001
> 1.9	34.5		17.4	
SCCRR (%)				
= 100	75.4	< 0.001	69.7	< 0.001
< 100	33.3		20.8	

*Radiological characteristics

On multivariate analysis, TD_pre_, TD_post_, and SCC_post_ were identified as independent predictors of OS. The OS of patients with TD_pre_ ≤ 4.4 cm was significantly better than that of patients with > 4.4 cm [Hazard ratio (HR) = 0.373, 95% confidence interval (CI): 0.155–0.898, *P* = 0.028]; the OS of patients with TD_post_ ≤ 2.4 cm was better than that of patients with > 2.4 cm (HR = 0.376, 95% CI 0.198–0.715, *P* = 0.003). The OS of patients with SCC_post_ ≤ 1.9 μg/L was better than that of patients > 1.9 μg/L (HR = 0.374, 95% CI 0.207–0.677, *P* = 0.001) (Table 3).

**Table 3:** Multivariate analysis of OS and PFS

Variable		Multivariate analysis		
		HR	95% CI	*P*
OS	TD_pre_ (cm)	0.373	0.155–0.898	0.028
	≤ 4.4/ > 4.4			
	TD_post_ (cm)	0.376	0.198–0.715	0.003
	≤ 2.4/ > 2.4			
	SCC_post_ (µg/L)	0.374	0.207–0.677	0.001
	≤ 1.9/ > 1.9			
PFS	TVRR (%)	2.998	1.739–5.171	< 0.001
	< 80.1/ ≥ 80.1			
	SCC_pre_ (µg/L)	0.563	0.325–0.977	0.041
	≤ 11.4/ > 11.4			
	SCC_post_ (µg/L)	0.253	0.143–0.447	< 0.001
	≤ 1.9/ > 1.9			

#### Analysis of PFS

On univariate analysis, SCC-Ag, FIGO staging, and chemotherapy cycles were all prognostic factors for PFS. TD_pre_ ≤ 4.4 cm group showed a significantly better 5-year PFS rate than TD_pre_ > 4.4 cm group (80.7% vs 52.2%, *P* < 0.001). TV_pre_ ≤ 45.71 cm^3^ group had better 5-year PFS than TV_pre_ > 45.71cm^3^ group (78.1% vs 50.0%, *P* < 0.001). The 5-year PFS of TD_post_ ≤ 2.4 cm group and TD_post_ > 2.4 cm group were 79.5% and 39.5%, respectively (*P* < 0.001). The 5-year PFS of TV_post_ ≤ 10.45 cm^3^ group and TV_post_ > 10.45 cm^3^ group was 78.1% and 36.4%, respectively (*P* < 0.001). The 5-year PFS of TVRR ≥ 80.1% group and TVRR < 80.1% group was 79.2% and 41.0%, respectively (*P* < 0.001). The 5-year PFS of SCC_pre_ ≤ 11.4 μg/L group and SCC_pre_ > 11.4 μg/L group was 73.1% and 43.1%, respectively (*P* < 0.001). The 5-year PFS of SCC_post_ ≤ 1.9 µg/L group and SCC_post_ > 1.9 µg/L group was 70.6% and 17.4%, respectively (*P* < 0.001). The 5-year PFS in the SCCRR 100% group and SCCRR < 100% group was 69.7% and 20.8%, respectively (*P* < 0.001) (Table 2).

On Cox regression multivariate analysis, TVRR, SCC_pre_, and SCC_post_ were identified as independent predictors of PFS. Patients with TVRR ≥ 80.1% showed obvious PFS benefit (HR = 2.998, 95% CI 1.739–5.171, *P* < 0.001). The PFS of patients with SCC_pre_ ≤ 11.4 μg/L was significantly better than that of patients with SCC_pre_ ≤ 11.4 μg/L (HR = 0.563, 95% CI 0.325–0.977, *P* = 0.041). The PFS of patients with SCC_post_ ≤ 1.9 μg/L was also better than that of patients with SCC_post_ > 1.9 μg/L (HR = 0.253, 95% CI 0.143–0.447, *P* < 0.001) (Table 3).

### Correlation analysis between tumor parameters

**Fig. 3:**
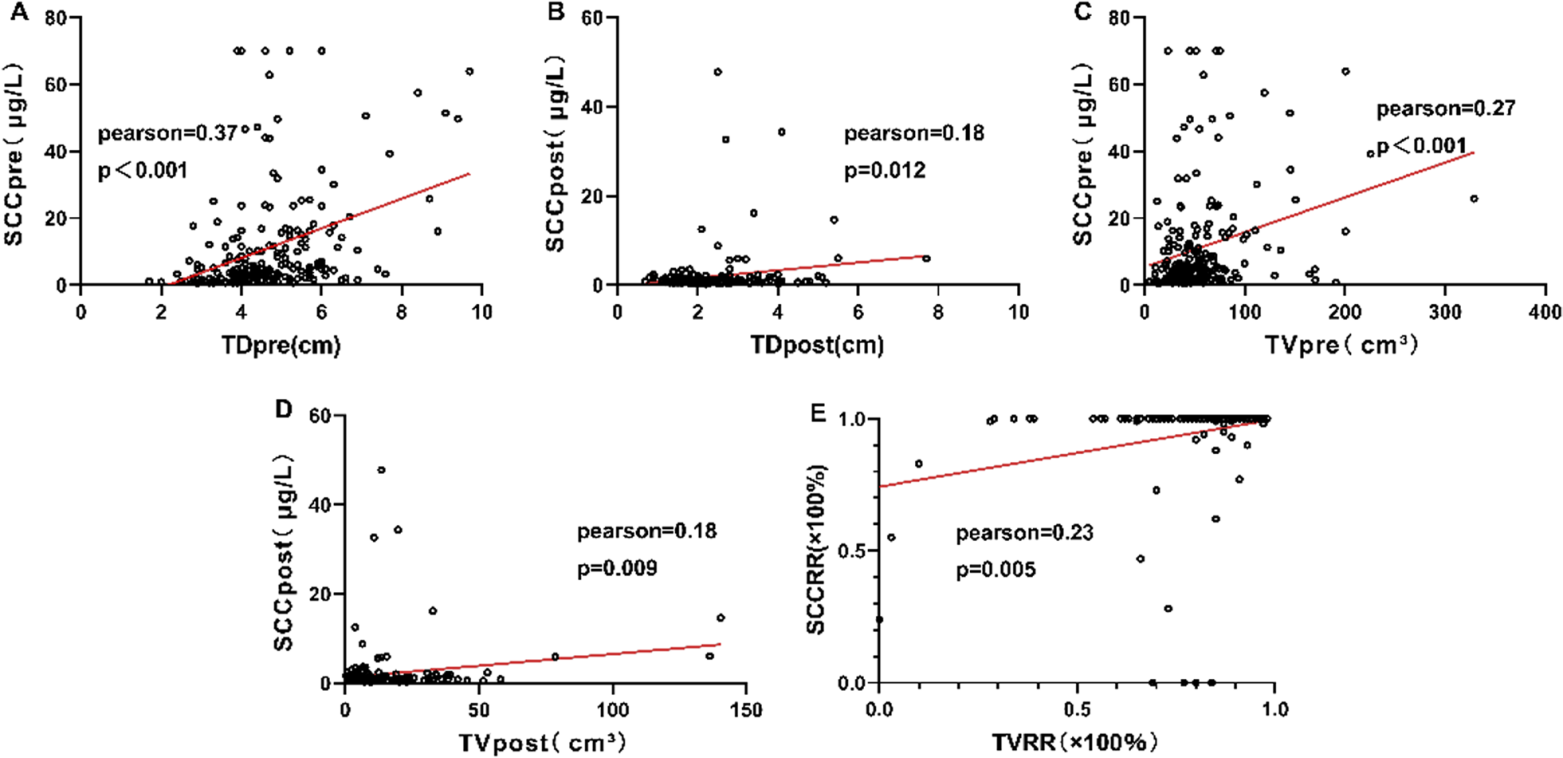
Results of correlation analysis: **a** Correlation between TD_pre_ and SCC_pre_; **b** TD_post_ and SCC_post_; **c** TV_pre_ and SCC_pre_; **d** TV_post_ and SCC_post_; **e** TVRR and SCCRR

We used a linear regression equation to further assess the correlation between tumor measurement parameters and SCC-Ag. TD_pre_ and SCC_pre_ showed the strongest correlation (Pearson = 0.37, *P* < 0.001). In addition, there was a certain correlation between TD_post_ and SCC_post_, between TV_pre_ and SCC_pre_, between TV_post_ and SCC_post_, and between TVRR and SCCRR (Fig. 3).

## Discussion

In this study, we investigated the prognostic value of tumor measurement parameters and SCC-Ag changes in patients with LACC. The study found that TD_pre_, TD_post_, TV_pre_, TV_post_, TVRR, SCC_pre_, SCC_post_, and SCCRR were all prognostic factors for CC. With the advances in imaging and radiotherapy technology, exploring the prognostic relevance of tumor diameter, volume, TVRR, and other measurement parameters in patients with cervical cancer is a key imperative. Lee et al. [17] conducted a retrospective study of 40 patients with CC. They found that pre-radiotherapy tumor volume > 55 cm^3^, tumor diameter during radiotherapy > 4 cm, and TVRR < 90% groups showed significantly poor PFS (5-year PFS: 69.7% vs 94.4%; 47.1% vs 88.0%; 61.3 vs 93.3%, respectively; *P* < 0.05). Ryu et al. [19] found that pre-treatment and post-treatment SCC-Ag values can predict the therapeutic efficacy and survival outcomes of patients with CC. In their study, SCC_pre_ > 1.86 µg/L and SCC_post_ > 0.9 µg/L groups had a longer median disease-free survival (DFS) than the respective control groups (median DFS: 132 months vs 148.5 months and 108 months vs. 147.5 months, respectively). The findings of Lee et al. and Ryu et al. indicated the prognostic value of tumor volume-related parameters and SCC-Ag in patients with CC. Therefore, it is worth further exploring the prognostic relevance of these indices. We used the 3D Slicer software system to accurately measure and calculate the pre- and post-treatment tumor parameters of each patient. In addition, we collected the SCC_pre_ and SCC_post_ values of each patient and calculated the SCCRR. Statistical analysis provided more robust data to identify the relevant prognostic factors of CC in order to guide clinical treatment.

Studies have shown considerable inter-individual variability with respect to the initial pre-treatment tumor volume and post-treatment residual volume. Currently, the optimal values of tumor diameter, volume, and TVRR are not clear, and no studies have identified the best time-point to measure the related parameters during treatment [20]. In our study, we performed ROC curve analysis to determine the optimal cut-off values of tumor measurement parameters and SCC-Ag. After adjusting for age, stage, and other prognostic factors, we found that TD_pre_ and TD_post_ were independent predictors of OS, while TVRR was an independent predictor of PFS. In a multicenter study [21], TD_pre_ > 6 cm (*P* = 0.0024) was an independent prognostic factor for LACC. However, in our study, the optimal TD_pre_ cut-off value was 4.4 cm. We also found that patients with TD_pre_ > 4.4 cm had poorer 5-year OS and 5-year PFS rates (58.3% vs. 84.1% and 52.2% vs. 80.7%, respectively; *P* < 0.001). Despite the different cut-off values of the parameters selected in each study, TD_pre_ was identified as an important factor affecting the prognosis of CC. The current FIGO staging includes TD_pre_ = 4 cm as one of the staging standards for IB and IIA stages, which is similar to the optimal cut-off level identified in our study.

However, can we also determine the optimal TD_post_ cut-off value or reference range? In the study by Lee et al. [22], TD_post_ = 1.8 cm was identified as the optimal cut-off value on ROC curve analysis. The 5-year OS and PFS in the TD_post_ ≤ 1.8 cm group and the control group was 96.2% vs 81.8% and 85.5% vs 58.8%, respectively (*P* < 0.05). In the present study, TD_post_ = 2.4 cm was the optimal cut-off value. The results suggest that patients with TD_post_ ≤ 2.4 cm have better 5-year OS and PFS (84.3% vs 44.7% and 79.5 vs 39.5%, respectively; *P* < 0.001). Moreover, it was an independent predictor of OS.

In this study, TVRR was found to be an important determinant of OS and PFS. Moreover, it was an independent predictor of PFS. The optimal cut-off value of TVRR was 80.1%. The 5-year OS and PFS were significantly better in patients with TVRR ≥ 80.1% (84% vs 46.2% and 79.2 vs 41%, respectively; *P* < 0.001). In the study by Lee et al. [23], TVRR was an independent predictor of OS (HR = 3.435, 95% CI 1.062–11.106, *P* = 0.039), and the 5-year OS rate in the TVRR > 87% group was significantly greater than that in the control group (96.5% vs 78%, *P* = 0.0003). Lee et al. [17] found that patients with TVRR ≥ 90% had better 5-year PFS (93.3% vs 61.3%, *P* = 0.031). The differences in the study population and the analysis time-points do not permit a direct comparison of the results of various studies. Nonetheless, all studies have identified the prognostic relevance of TVRR in CC. The smaller the TVRR, the worse is the prognosis of patients. Therefore, we also discuss the reasons why TVRR affects the prognosis of CC. Tewari et al. [22] found that chemotherapy can improve the tumor sensitivity to radiotherapy in patients undergoing concurrent chemoradiation, while radiotherapy further improves the local control rate. Some researchers found that the shrinkage of tumor after chemotherapy directly reflects the sensitivity of tumor cells to chemotherapy to a certain extent. Lack of obvious tumor shrinkage implies poor tumor control. In this setting, there is a likelihood of micrometastasis in the circulatory system, which may eventually lead to recurrence or metastasis [24–27].

In addition, we also assessed the prognostic value of SCC-Ag in patients with CC. SCC-Ag is used as one of the diagnostic markers for squamous cell carcinoma. SCC-Ag can be used to judge the prognosis of CC and predict the possibility of recurrence [15]. At present, the critical level for defining normal SCC-Ag is also different between different studies (≤ 1.5 μg/L vs ≤ 2.0 μg/L) [28, 29]. SCC-Ag cut-off value in our study was 2.0 µg/L. It should be noted that SCC-Ag often needs to be used in combination with other factors to evaluate the prognosis of CC. Choi et al. [11] retrospectively analyzed 304 patients with CC who received concurrent chemoradiation. They found that SCC_pre_ = 4.0 µg/L was the best cutoff value, and the results showed that the 3-year RFS rates (56.6% vs 80.2%, *P* < 0.001) and OS rates (72.1% vs 86.8%, *P* = 0.005) of patients with SCC_pre_ ≥ 4 μg/L were significantly lower than those of patients with SCC_pre_ < 4 μg/L. In our study, the optimal cut-off value of SCC_pre_ was 11.4 μg/L, and Cox regression multivariate analysis identified SCC_pre_ as an independent predictor of PFS. In addition, we observed a significant positive correlation between SCCpre and TD_pre_ (Pearson = 0.37, *P* < 0.001). The results of this study also suggest that SCC_pre_ can be used to assess tumor burden and predict prognosis.

We believe that the SCC_post_ value may play an important role in the decision-making of follow-up treatment of CC [19, 30, 31]. Kawaguchi et al. [30] evaluated the SCC-Ag value at 1 month after treatment. They found that the prognosis of patients with SCC_post_ < 1.15 μg/L was significantly better than that of patients with SCCpost ≥ 1.15 μg/L (3-year OS: 90.7% vs 36.6%; 3-year PFS: 74.7% vs 19.5%, *P* < 0.001). Our study also identified SCC_post_ as an important factor affecting prognosis. The 5-year PFS in the SCC_post_ ≤ 1.9 µg/L group and SCC_post_ > 1.9 µg/L group was 70.6% and 17.4%, respectively (*P* < 0.001). In the study by Ryu et al. [19], SCC_post_ = 0.9 µg/L was the optimal cut-off value for predicting tumor recurrence. SCC_post_ was an independent predictor of DFS. Although the best cut-off value of SCC_post_ was different in each study, all studies have identified the prognostic value of SCC_post_; patients who had SCC_post_ higher than normal had poor prognosis.

In addition, we also found that the SCC-Ag of most patients with CC was significantly reduced after concurrent chemoradiation. Therefore, it is also very important to evaluate the predictive value of SCCRR for therapeutic efficacy. Markovina et al. [32] found that SCC-Ag gene knockout increased the radiosensitivity of CC cells cultured in vitro; this showed that SCCRR can indeed increase the radiotherapeutic efficacy. Therefore, many scholars believe that SCCRR can be used to predict the tumor response rate or survival of CC patients after receiving chemoradiation [17, 32]. In the study by Lee et al. [22], SCCRR showed an independent association with OS (*P* = 0.003); the 5-year OS of patients with SCCRR ≤ 93.3% and SCCRR > 93.3% was 74.9% and 95.4%, respectively (*P* < 0.0001). We found that SCCRR was one of the prognostic factors influencing the OS and PFS of patients with LACC. Indeed, there was also a certain correlation between SCCRR and TVRR. However, since the correlation between SCCRR and TVRR was not very strong (Pearson = 0.23, *P* = 0.005), further studies are required to obtain more definitive evidence.

There are several limitations in this retrospective study. Firstly, the nature of a retrospective study certainly served as an inherited and fundamental limitation. Secondly, the study lacks of a verification cohort. Finally, we didn’t perform the same prognostic analysis by subgroups stratifying by stage of disease.This will be the direction of our future research.

## Conclusions

In this study, TD_pre_, TD_post_, and SCC_post_ were independent predictors of OS of patients with CC. TVRR, SCC_pre_, and SCC_post_ were independent predictors of PFS. These tumor parameters and level of SCC-Ag were very good predictors of tumor response rate during treatment.

## Data Availability

The data used or analyzed during the current study are available from the corresponding author on reasonable request.
